# Effect of Astragaloside IV on Neural Stem Cell Transplantation in Alzheimer's Disease Rat Models

**DOI:** 10.1155/2016/3106980

**Published:** 2016-02-23

**Authors:** Hu Haiyan, Yang Rensong, Jin Guoqin, Zhang Xueli, Xia Huaying, Xu Yanwu

**Affiliations:** Department of Biochemistry, Shanghai University of Traditional Chinese Medicine, 1200 Cailun Road, Shanghai 201203, China

## Abstract

Stem cell-based therapy is a promising treatment strategy for neurodegenerative diseases such as Alzheimer's disease (AD). However, the mechanism underlying the maintenance of renewal and replacement capabilities of endogenous progenitor cells or engrafted stem cells in a pathological environment remains elusive. To investigate the effect of astragaloside IV (ASI) on the proliferation and differentiation of the engrafted neural stem cells (NSCs), we cultured NSCs from the hippocampus of E14 rat embryos, treated the cells with ASI, and then transplanted the cells into the hippocampus of rat AD models.* In vitro* experimentation showed that 10^−5^ M ASI induced NSCs to differentiate into *β*-tubulin III^+^ and GFAP^+^ cells. NSCs transplantation into rat AD models resulted in improvements in learning and memory, especially in the ASI-treated groups. ASI treatment resulted in an increase in the number of *β*-tubulin III^+^ cells in the hippocampus. Further investigation showed that ASI inhibited PS1 expression* in vitro* and* in vivo*. The high-dose ASI downregulated the Notch intracellular domain, whereas the low-dose ASI increased Notch-1 and NICD. In conclusion, ASI treatment resulted in improvements in learning and memory of AD models by promoting NSC proliferation and differentiation partly through the Notch signal pathway.

## 1. Introduction

The most common cause of dementia is Alzheimer's disease (AD), which is a neurodegenerative disorder characterized by cognitive and memory deficiencies. The presence of intracellular neurofibrillary tangles and extracellular amyloid deposits in the brain are the hallmarks of AD [1]. The progressive accumulation of *β*-amyloid peptide (A*β*) is widely believed to trigger a cascade of events that contribute to the progression of AD. Antiamyloid therapies are considered to be the most promising treatment for AD, although its efficacy in clinical practice appears to be limited. Emerging evidence has indicated that altered neurogenesis in the adult hippocampus represents an early critical event in the course of AD. Several studies have reported that a decline in neurogenesis within the subgranular zone (SGZ) and the subventricular zone (SVZ) is involved in cognitive impairment that is linked to aging and neurodegenerative disorders [2, 3]. A study of neurogenesis in postmortem human brains has shown an increase in the number of immature neurons in the hippocampus of AD [4]. On the other hand, no alterations in DG neurogenesis have been observed in presenile AD cases [5]. These findings suggest that there might be a self-compensating mechanism that replaces lost neurons and the pathogenic brain environment has impeded this particular mechanism. Neural stem cell (NSC) replacement or the maintenance of an appropriate microenvironment for successful neurogenesis is currently considered as a potential therapeutic approach for counteracting age-associated memory dysfunction. Actually, the number of engrafted cells which could indeed translate into effective therapies for currently intractable disorders is still a challenge on the road.

In recent years, several studies have examined the effects of a single compound or a mixture of traditional Chinese medicine on improving neurogenesis or regulating NSCs microenvironment [6, 7]. Astragaloside IV (ASI) is a small molecular saponin found in* Astragalus membranaceus*, which is a widely used herb in Chinese medicine. This saponin possesses various pharmacological activities such as anti-inflammation [8], antioxidative [9–11], or anti-infarction effects [12]. Moreover, ASI also imparts a neuroprotective effect by promoting axonal regeneration and reconstruction of neuronal synapses [13]. In the present study, we investigated whether ASI treatment could promote the proliferation and differentiation of engrafted NSCs and ameliorate cognitive impairment by amyloid-*β* intracerebral injection of AD rat models.

## 2. Materials and Methods

### 2.1. Drugs

ASI was purchased from the National Institute for Food and Drug Control in China, dissolved in dimethyl sulfoxide to create a stock solution of 0.01 M, and diluted in saline to various working concentrations.

### 2.2. Cells

Hippocampus cells were isolated from 14-day Sprague-Dawley rat embryos. The cells were isolated and propagated as described by Reynolds and Weiss [25]. Single cells were cultured in basal serum-free medium consisting of DMEM/F12 (Gibco, Carlsbad, CA, USA), 10% B27 (Invitrogen, Carlsbad, CA), 0.006 pg/mL progesterone (Sigma, St. Louis, MO), 9.6 pg/mL putrescine (Sigma, St. Louis, MO), 10 ng/mL ITSS (Roche Diagnostics, Mannheim, Germany), 2 ng/mL heparin (Sigma, St. Louis, MO), 0.05 ng/mL EGF (Peprotech, Rocky Hill, NJ), and 5 ng/mL FGF2 (Peprotech, Rocky Hill, NJ). The cultures were maintained in a humidified chamber set at 37°C, with 5% CO_2_ air. Half the medium was changed every 3 days.

### 2.3. ASI Treatment and MTT Assay

The cells were transferred to 96-well poly-L-lysine coated plates and treated with 10^−5^, 10^−6^, and 10^−7^ M ASI for 3 days. Positive controls consisted of 1% fetal bovine serum-treated NSCs. The viability of cells was evaluated by using the MTT assay. After the various experimental treatments, and at the end of the respective incubation periods, 100 *μ*L of the MTT solution was added to each well, followed by incubation of the plates for 4 h at 37°C in a CO_2_ incubator. The reaction was terminated by removal of the media and adding 200 *μ*L of dimethylsulphoxide. The levels of reduced MTT were determined by measuring the absorbance at a wavelength of 570 nm using a plate reader (Biotek Synergy two, Winooski, VT). Each experiment was independently repeated 3 times using a minimum of 3 plates/experiment.

### 2.4. Animals and Treatments

Eight-week-old Sprague-Dawley rats were purchased from the Shanghai Laboratory Animal Research Centre. Rats were randomly divided into six groups: control, vehicle, model, DH, uninduced NSCs transplants (TP), and ASI induced NSCs transplants (TP-ASI) (Table 1). The models were produced by bilateral intrahippocampal injection of 5 *μ*g of A*β* to each side under anesthesia using 10% chloral hydrate. Approximately 48 h prior to transplantation, undifferentiated NSCs were labeled with 5-bromo-2′-deoxyuridine (BrdU, Sigma-Aldrich), then the cultured NSCs were induced by 10^−5^ M ASI for 24 h, and then about 10^5^ cells were transplanted bilaterally into the hippocampus one week after model production. The rats were then intraperitoneally injected with 5 mg/kg/day of ASI for 4 weeks. Vehicle rats were injected simultaneously with solvents using the same routes. Donepezil hydrochloride (1.0 mg/kg/day, intragastric, Sigma, St. Louis, MO) was used as positive control. On the last week, rats were tested for their learning and memory ability by using the Morris water maze test. Some of the animals in each group were then deeply anaesthetized with chloral hydrate, prefixed with an intracardiac infusion of 4% paraformaldehyde, followed by the isolation of their brains and the preparation of serial 5 *μ*m thick coronal frozen sections for immunohistochemical examination. The rest of the animals were sacrificed and the brains were immediately stored at −70°C. All the animal experiments were conducted according to the protocol approved by the Animal Care and Use Committee of the Shanghai University of Traditional Chinese Medicine (Protocol #11051).

### 2.5. Morris Water Maze Test

Four weeks after transplantation, the rats (*n* = 18) were trained on two trials per day and for a total of 6 days in the Morris water maze. The water maze consisted of a circular black pool measuring 1.20 m in diameter and 0.45 m in height. The pool was filled with water (21 ± 1°C) to a depth of 27 cm. A circular escape platform (8 cm in diameter) was placed just below the water surface and the surrounding cues were used as sample. Four positions around the edge of the tank were arbitrarily designated north (N), south (S), east (E), and west (W), which provided four alternative start positions. The hidden platform remained in the same quadrant during training, whereas the start positions (N, S, E, or W) were randomized across trials. The rats were allowed up to 70 s to locate the escape platform, and their escape latency was automatically recorded by a DigBehv system (Shanghai Jiliang Software Technology, Shanghai, China). On the last trial of the last training day, rats received a single probe trial, during which the escape platform was removed from the tank and the percentages of platform quadrant were recorded over a span of  70 s while rats searched for the missing platform and the percent of time in each quadrant by using the DigBehv system.

### 2.6. Immunostaining

Cells were cultured in 10^−5^ M ASI for three days. The cells or coronal frozen rat brain sections were fixed in 4% paraformaldehyde, treated sequentially with H_2_O_2_, Triton X-100, and bovine serum albumin, and then incubated with primary antibodies at the following concentrations: nestin, 1 : 200 (Cell signal Technology, Danvers, MA); *β*-tubulin III (Tuj1), 1 : 500 (Epitomics, Burlingame, CA); GFAP, 1 : 300 (Cell signal Technology, Danvers, MA); and BrdU, 1 : 300 (Cell signal Technology, Danvers, MA). The sections were incubated with the corresponding primary antibodies at 4°C overnight. After thorough washing in PBS, the sections were further incubated with secondary antibodies. Fluorescent images were captured on an Axiovert 40, Zeiss.

### 2.7. Western Blot Analysis

Cells were cultured in 10^−5^ M ASI for three days prior to harvesting. Brain tissues were obtained from euthanized rats after completion of the behavioral experiments. For western blot analyses, 50 *μ*g of protein was denatured in a sample buffer for 5 min at 100°C, and proteins were separated on SDS-polyacrylamide gels. The proteins were transferred onto a polyvinylidene difluoride (PVDF) membrane, blocked with skim milk, and incubated overnight at 4°C in the corresponding primary antibody [i.e., Notch-1, 1 : 2,000, Cell signal Technology; NICD, 1 : 1000, Cell signal Technology; PS-1, 1 : 2,000, Chemicon; *β*-actin, 1 : 1,000, Santa Cruz; and *β*-tubulin III (Tuj-1), 1 : 1,000]. After incubation, the slides were washed, incubated with their respective secondary antibodies, and finally color-developed by using ECL reagents (Thermo, Waltham, MA). Densitometric quantification was performed with an image analyzer (Gel Doc 2000, Bio-Rad). The gray values were normalized to that of the *β*-actin antibody.

## 3. Results

### 3.1. Intermediate- and Low-Dose ASI Increase the Rate of Proliferation of Rat NSCs* In Vitro*

**Figure 1:**
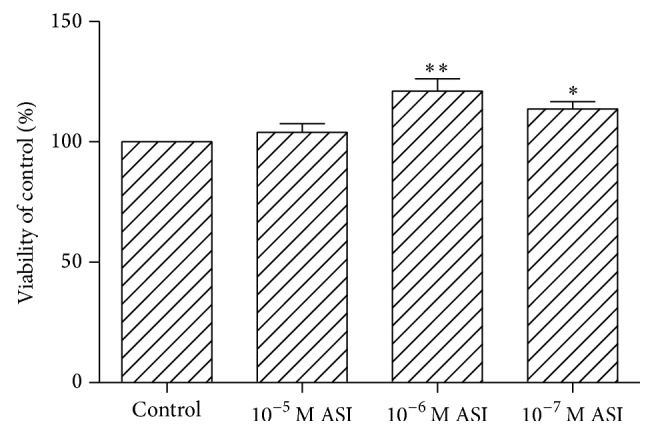
Viability of neural stem cell treated by 10^−5^, 10^−6^, or 10^−7^ M ASI for 3 days. Values are expressed as means ± SEM from 8 independent experiments. ^*∗*^ *p* < 0.05 and ^*∗∗*^ *p* < 0.01 compared to control statistic by one-way ANOVA followed by Bonferroni's Multiple Comparison test.

The MTT assay showed that the treatment of NSCs with 10^−6^ or 10^−7^ M ASI for 3 days significantly increased cell viability. On the other hand, the application of a high concentration (10^−5^ M) of ASI did not result in any detectable change in the rate of cell proliferation (Figure 1).

### 3.2. High-Dose ASI Induces NSC Differentiation* In Vitro*

**Figure 2:**
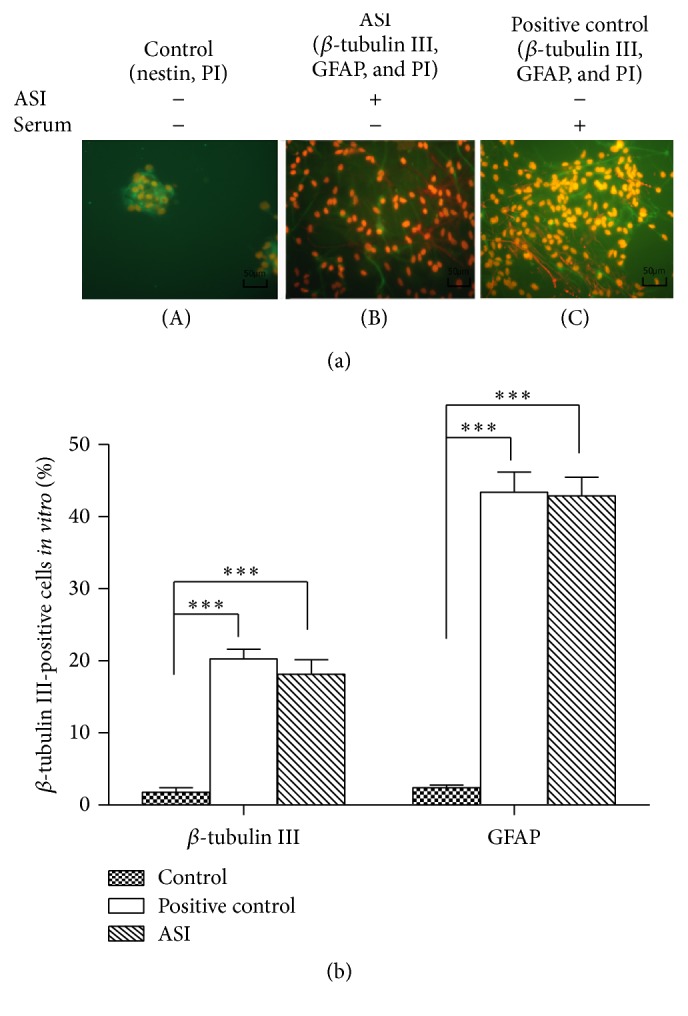
(a) (A) Immunocytochemistry of* in vitro* cultured NSCs. (B)~(C) 10^−5^ M ASI induced the NSC to differentiate into neuron (red: *β*-tubulin III) and glial cells (green: GFAP). (b) Quantification of cultured cells differentiation is expressed as mean ± SEM (*n* = 8), ^*∗∗∗*^ *p* < 0.001, statistic by one-way ANOVA followed by Bonferroni's Multiple Comparison test.

Immunocytochemical analysis showed that treatment with 10^−5^ M ASI induced 18.13 ± 2.02% and 42.88 ± 2.62% of the cultured NSCs to differentiate into neurons and astrocytes, respectively. These ratios were similar to those observed in the positive control group, which were 20.25 ± 1.36% and 43.38 ± 2.80%, respectively (Figure 2).

### 3.3. Effect of ASI on Learning and Memory of AD Rat Models

**Figure 3:**
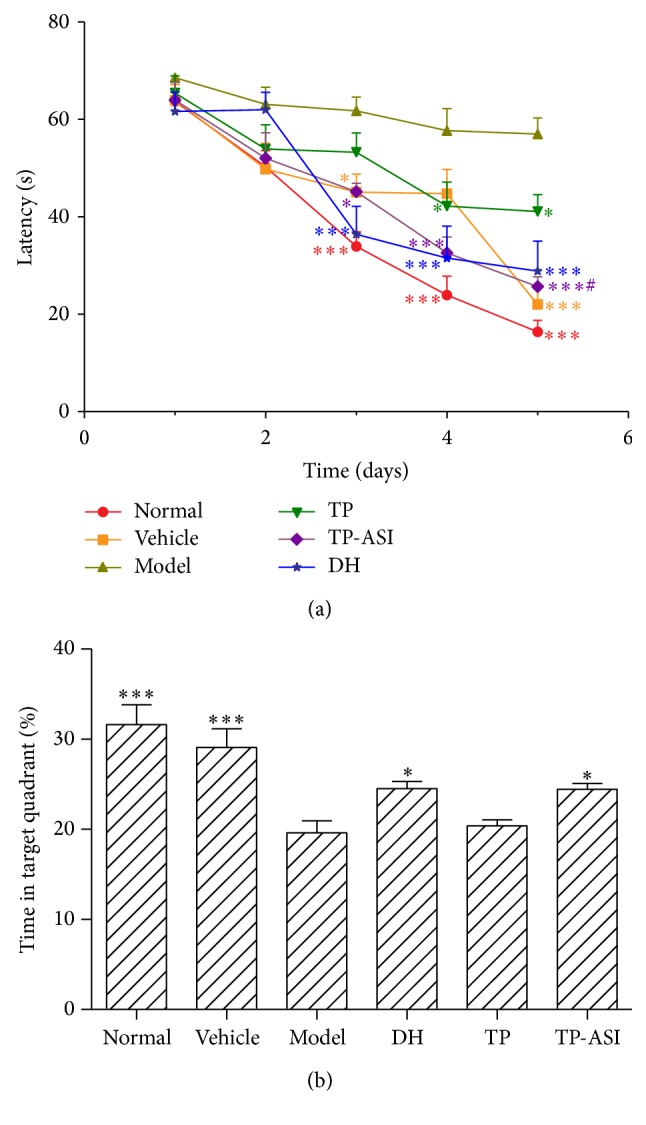
Morris' water maze acquisition and probe performance in different groups of rats. (a) showed acquisition latencies to reach the hidden platform during 5 days of trail. The latencies in TP-ASI group were shorter than model on days 3, 4, and 5. Statistic by two-way ANOVA followed by Bonferroni's posttests to compare replicate means by row. (b) showed probe trail expressed in percent time in target quadrant. Rats of TP-ASI and DH group spent significantly more time in target quadrant than model. Statistic by one-way ANOVA followed by Bonferroni's Multiple Comparison test. Values were expressed as means ± SEM. ^*∗*^ *p* < 0.05 and ^*∗∗∗*^ *p* < 0.001 versus model; ^#^ *p* < 0.05 versus TP; *n* = 18.

The rats were randomly assigned to six different treatment groups: control, vehicle, model, donepezil (DH; 1.0 mg/kg/day, intragastric), uninduced NSCs transplants (TP), and ASI induced NSCs transplants (TP-ASI). We first assessed spatial memory abilities to engage in the Morris water maze task, which is a cognitive paradigm wherein AD mice are known to be impaired. In a hidden platform trial, when daily trials were plotted, latencies were not different among all groups on the first two days (Figure 3(a)). The latencies of the control, vehicle, TP-ASI, and DH groups were relatively shorter than that observed in the model from day 3 to day 5. The latency of TP at days 4 and 5 was significantly shorter than that of the model group. Comparison of the TP-ASI and TP groups showed great significant difference. The probe trial involved the removal of the hidden platform and measurement of the amount of time spent in the target platform (Figure 3(b)). The probe test confirmed the spatial memory impairment of the rat models. Rats from the TP-ASI group (24.35% ± 0.91) and the DH group (24.5% ± 1.08) spent significantly longer time than that observed in the model (19.63% ± 1.32) in the quadrant where the platform was located.

### 3.4. ASI Increased the Number of Immature Neurons in the AD Rat Brain

**Figure 4:**
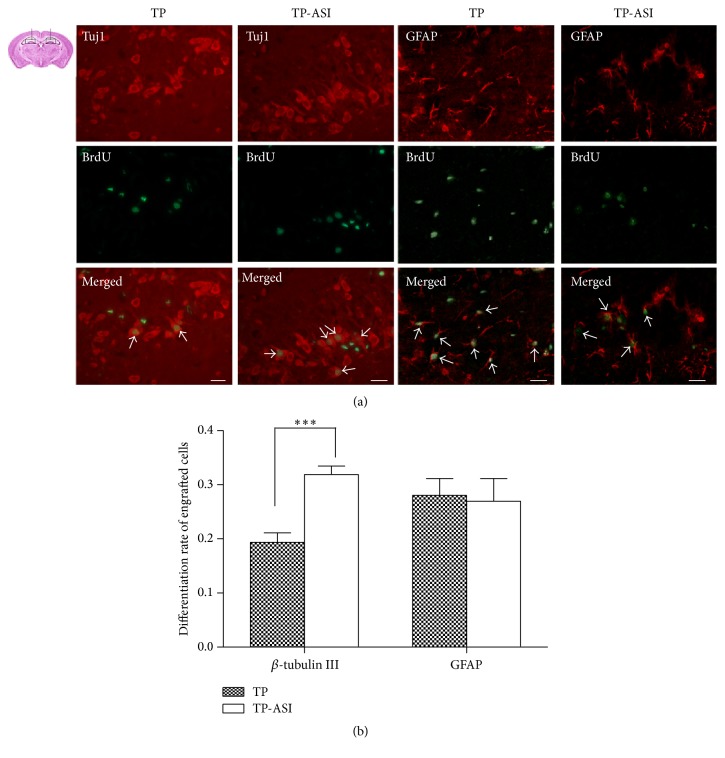
(a) Differentiation of transplanted NSCs into immature neurons (BrdU^+^/Tuj1^+^) and astroglial (BrdU^+^/GFAP^+^) in the DG at 5 weeks after engraftment. TP-ASI resulted in more neurons than TP. (b) Quantification of positive cells is expressed as mean ± SEM (*n* = 20), ^*∗∗∗*^ *p* < 0.001, statistic by *t*-test (scale bars: 10 *μ*m).

Immunohistochemical analysis showed that, 5 weeks after transplantation, BrdU-labeled cells which were derived from imbedding had spread across the entire hippocampus. Approximately 20.8 ± 1.70% of the engrafted cells differentiated into immature neurons (BrdU^+^/Tuj1^+^) in the TP group and the ratio increased to 31.9 ± 1.51% in the ASI TP-ASI group. The ratio of those differentiated into astrocytes (BrdU^+^/GFAP^+^) showed no changes between TP-ASI and TP (Figure 4).

### 3.5. ASI Induced the Upregulation of Notch-1 and the Downregulation of PS-1

**Figure 5:**
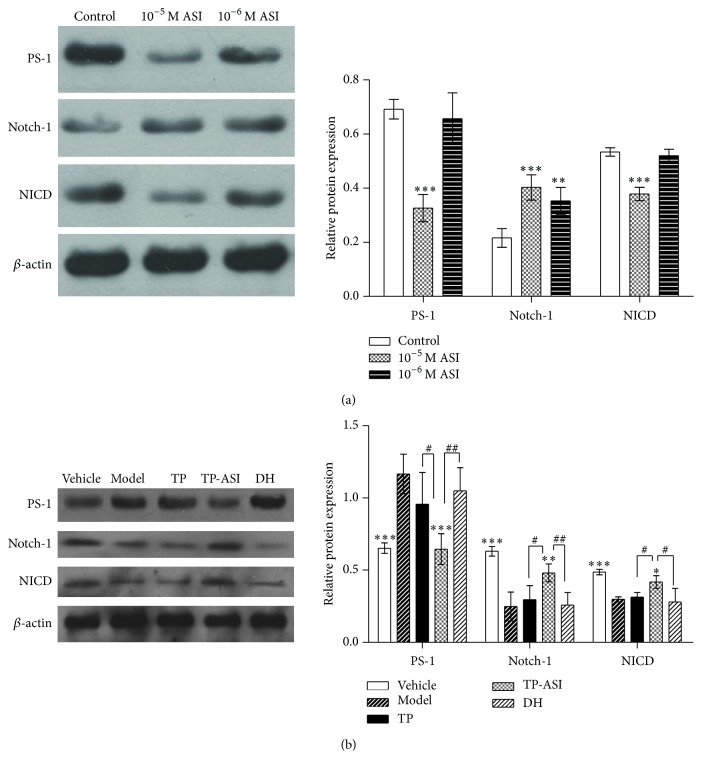
Effects of astragaloside IV on Notch pathway. (a) Western blots of PS-1, Notch-1, and NICD in cultured NSCs and their gray intensity analysis. (b) Western blots of PS-1, Notch, and NICD in treated rats' brain and their gray intensity analysis. All data are presented as means ± SEM. ^*∗*^ *p* < 0.05, ^*∗∗*^ *p* < 0.01, and ^*∗∗∗*^ *p* < 0.001 compared with model. ^#^ *p* < 0.05 and ^##^ *p* < 0.01 compared with TP-ASI, statistic by one-way ANOVA followed by Bonferroni's Multiple Comparison test.

The effect of ASI on the Notch signaling pathway in NSCs was evaluated. Western blotting of cultured cells showed that 10^−5^ M ASI (*p* < 0.001, *n* = 5) and 10^−6^ M ASI (*p* < 0.01, *n* = 5) resulted in the upregulation of Notch-1 (Figure 5(a)). We also tested the expressions of the Notch intracellular domain, NICD, which was the active form of the* Notch-1* gene that is cleaved by *γ*-secretase. Downregulation of NICD was observed in cells treated with 10^−5^ M ASI (*p* < 0.001, *n* = 5), whereas minimal changes in the level of expression were observed in the 10^−6^ M treatment group. Subsequently, we examined the active subunit of the *γ*-secretase complex, presenilin-1 (PS-1), which showed that 10^−5^ M ASI significantly reduced the expression of PS-1 (*p* < 0.001, *n* = 5).

The level of PS-1 protein in the AD models was higher than that in the vehicle (*p* < 0.001), whereas transplanting the ASI induced NSCs resulted in PS-1 downregulation (*p* < 0.001; Figure 5(b)). Notch and NICD expression levels were higher in the TP-ASI group compared to the model (*p* < 0.01 and *p* < 0.05) and the TP group (*p* < 0.05, resp.). DH did not change the three proteins compared to the model.

## 4. Discussion

The present study demonstrated the effects of ASI on the proliferation and differentiation of NSCs* in vitro* and* in vivo*. ASI-pretreated NSCs transplanted into the brain showed an improvement in spatial learning and memory abilities. Further studies were focused on the role played by presenilin-1 and its target protein Notch-1 on the proliferation and differentiation effects of ASI.

First, we tested the effect of different doses of ASI on cultured rat NSCs. The results showed that 10^−7^ M and 10^−6^ M ASI imparted a slight increase in the rate of proliferation. On the other hand, 10^−5^ M ASI did not increase the cell proliferation rate but induced stem cell differentiation into nestin^+^ and GFAP^+^ cells, as indicated by the immunostaining (Figure 2). The differentiation ratios were similar to those of the positive controls using 1% serum. The results were also in agreement with the findings of Cheng et al. [13], which demonstrated that astragaloside plays a dual role in peripheral nerve regeneration, wherein a low dose induced a higher rate of regeneration, whereas a high dose did not. Other reports have described that Huangqi or its extract induced differentiation of bone marrow mesenchymal stem cells [14].

The engraftment potential of NSCs [15] has sparked interest in its application to treatment modalities for neurodegenerative diseases [16, 17]. Kim and colleagues transplanted ChAT-overexpressing NSCs into the right ventricle of AF64A-lesioned AD rat models, which led to the recovery of learning and memory impairment. However, in theory, although a large supply of fetal cells are obtained, these have a low level of capability to participate in CNS repair in the presence of degenerative diseases or traumatic injury, thus overshadowing its therapeutic benefit [18]. Based on these previous findings, there have been extensive efforts to increase the survival and pluripotency of engrafted NSCs. In the present study, we investigated the effect of ASI on engrafted NSCs* in vivo*. We first pretreated the cultured NSCs with ASI and then implanted the cells into the brain of an AD rat model, which were intraperitoneally injected with ASI for 4 weeks to sustain efficacy. Donepezil hydrochloride was used as positive control. In the spatial acquisition task, the latencies to locate the submerged platform were no different among the groups on the first 2 days. The latencies of the model group were significantly longer than those observed in the other groups during the next 3 days. The animals of the TP-ASI group showed an improvement in the time required to find the platform on days 3, 4, and 5. The short latency of the TP-ASI group appeared one day earlier than that of the TP group compared to the model and was significantly shorter on day 5 than that of the TP group. To assess the strength of spatial learning, we performed a probe trial in which the hidden platform was removed and the amount of time spent in the former region of platform, namely, percent time in the target quadrant, was measured. The probe tests confirmed the mending of spatial memory impairment in the TP-ASI group. These data suggest that NSC engraftment improves the learning and memory capacity of rats with dementia. Transplantation of ASI-treated NSCs showed a more encouraging effect than that observed using transplanted cells alone.

The cells used in the transplantation were BrdU-labeled. It was observed that the engrafted NSCs migrated throughout the hippocampus. Some of the transplanted cells differentiated into immature neurons (*β*-tubulin III^+^/BrdU^+^) and astroglial (GFAP^+^/BrdU^+^). There were more *β*-tubulin III-positive cells in the TP-ASI group than in the TP group. On the other hand, no differences in the number of GFAP-positive cells were observed between the TP and the TP-ASI groups. These data suggested that ASI has the ability to increase the engrafted NSCs differentiating into neuron* in vivo*.

To elucidate the mechanism of ASI in NSCs' proliferation and differentiation, we tested its effect on the Notch signal pathway, which is one of the major signal systems that govern the neurogenesis. The Notch signaling pathway was found to play a critical role in the maintenance and renewal of neural progenitor cells (NPCs). Activation of the pathway was sufficient to maintain NPCs in its proliferative state, whereas loss-of-function mutations in the critical components of the pathway caused precocious neuronal differentiation and NPC depletion. NSCs depleted in the early embryonic brains of RBP-J*κ* ^−/−^ or Notch-1^−/−^ mice and the active form of Notch-1 enhance the* in vitro* symmetric division of neural stem cells from the E14.5* PS1* ^−/−^ brain, which has self-renewal defects [19]. Recent studies have also shown that Notch-1 is downregulated in stroked or in aged brains [20, 21]. These findings confirm the importance of the Notch signaling pathway in stem cell replacement in pathological situations. One component of *γ*-secretase, presenilin-1 (PS-1), is required for the cleavage of the Notch intracellular domain (NICD) and PS-1 is also needed in the formation of A*β*. The NICD was translocated to the nucleus and bound to the transcription factor CSL (RBP-j), allowing it to participate in a complex activating its target genes [22], and eventually led to NPCs proliferation. Our previous research has found that ASI induced the differentiation of NSCs and affects the level of *γ*-secretase mRNA expression [23]. In the present study, we found that although both doses of ASI (10^−5^ M and 10^−6^ M) could increase Notch-1 expression, NICD generation did not elevate with the Notch-1 in the high-dose group. Further testing showed that the high-dose ASI inhibited PS-1 expression* in vitro*, whereas the low dose did not. So, although high-dose ASI elevates Notch-1, low level of PS-1 hinders its activation. Changes in NICD correspond well with the fate of cultured NSCs as being found in morphology observation, low-dose ASI promoted the cell proliferation, and high-dose ASI induced the cell differentiation. It suggests that ASI induces the proliferation and differentiation of NSCs by regulating the Notch pathway.* In vivo*, we observed that PS-1 expression increased in the A*β*-injected groups. Mere NSCs transplant could not resist this overexpression whereas ASI induced NSCs transplant (TP-ASI) decreased this particular change (Figure 4). These results proved that ASI could inhibit PS-1* in vivo*, too. TP-ASI could increase both Notch-1 and NICD significantly compared to the model and TP group. Simple engraftment of NSCs or intragastric DH did not result in significant changes in the two proteins. Although there were no significant differences between TP-ASI and DH group in behavioral tests, donepezil, which was a kind of acetylcholinesterase inhibitor, did not influence the Notch pathway. So it could not affect the NSCs' fate. On a long view, the efficacy of donepezil will decrease with gradually losing acetylcholine neuron in AD brain, whereas NSCs could replenish the damaged neurons in the long term. It is generally recognized that Notch signal pathway activation leads to the self-renewal of NSCs. But there are conflicts between Notch stimulus and NSCs differentiation in TP-ASI in our findings. This suggests the aforehand ASI treatment plays an important role in the fate of the engrafted NSCs. Since the distributions of ASI in cerebrospinal fluid are low [24], they cause the increase of NICD as low-dose ASI do* in vitro*. So we supposed that ASI would help to maintain the endogenous NSCs number through stimulating the Notch signal pathway. Further experiments should be carried out to explore details.

In conclusion, these data highlight the effect of ASI on transplanted NSCs on improving spatial learning and memory in an AD rat model and the possible mechanism of it. Data showed that ASI affects NSC proliferation or differentiation by using various doses. High concentration ASI reduces the level of PS-1 and inhibits Notch signal, whereas low concentration activates the Notch signal. The changes are integrated to make a more suitable neurogenic niche that might contribute to the dynamic, complex, and context-dependent process of neurogenesis.

## Figures and Tables

**Table 1:** Treatments of animals.

Treatments	Groups					
	Normal	Vehicle	Model	TP	TP-ASI	DH
A*β* intrahippocampal injection	—	Solvent	A*β*	A*β*	A*β*	A*β*
NSC transplant	—	Solvent	Solvent	NSC	ASI-NSC	Solvent
Drug treatment	—	Solvent	Solvent	Solvent	ASI	DH

## Abbreviations

ASI: Astragaloside IV
NICD: Notch intracellular domain
NSCs: Neural stem cells
SGZ: The subgranular zone
SVZ: The subventricular zone.

## References

[1] Hardy J., Selkoe D. J. (2002). The amyloid hypothesis of Alzheimer's disease: progress and problems on the road to therapeutics. *Science*.

[2] Rodríguez J. J., Jones V. C., Tabuchi M. (2008). Impaired adult neurogenesis in the dentate gyrus of a triple transgenic mouse model of Alzheimer's Ddisease. *PLoS ONE*.

[3] Kuhn H. G., Dickinson-Anson H., Gage F. H. (1996). Neurogenesis in the dentate gyrus of the adult rat: age-related decrease of neuronal progenitor proliferation. *The Journal of Neuroscience*.

[4] Jin K., Peel A. L., Mao X. O. (2004). Increased hippocampal neurogenesis in Alzheimer's disease. *Proceedings of the National Academy of Sciences of the United States of America*.

[5] Boekhoorn K., Joels M., Lucassen P. J. (2006). Increased proliferation reflects glial and vascular-associated changes, but not neurogenesis in the presenile Alzheimer hippocampus. *Neurobiology of Disease*.

[6] Ren Z. L., Zuo P. P. (2012). Neural regeneration: role of traditional Chinese medicine in neurological diseases treatment. *Journal of Pharmacological Sciences*.

[7] Si Y.-C., Li Q., Xie C.-E., Niu X., Xia X.-H., Yu C.-Y. (2014). Chinese herbs and their active ingredients for activating *xue* (*blood*) promote the proliferation and differentiation of neural stem cells and mesenchymal stem cells. *Chinese Medicine*.

[8] Zhang W.-J., Hufnagl P., Binder B. R., Wojta J. (2003). Antiinflammatory activity of astragaloside IV is mediated by inhibition of NF-*κ*B activation and adhesion molecule expression. *Thrombosis and Haemostasis*.

[9] Qiu L.-H., Xie X.-J., Zhang B.-Q. (2010). Astragaloside IV improves homocysteine-induced acute phase endothelial dysfunction via antioxidation. *Biological and Pharmaceutical Bulletin*.

[10] Wang S.-B., Qiu J.-F., Bai Q.-H. (2011). A study on protection of astragaioside IV about oxidative stress on PC12 cells induced by H_2_O_2_. *Chinese Pharmacological Bulletin*.

[11] He Y., Du M., Gao Y. (2013). Astragaloside IV attenuates experimental autoimmune encephalomyelitis of mice by counteracting oxidative stress at multiple levels. *PLoS ONE*.

[12] Luo Y., Qin Z., Hong Z. (2004). Astragaloside IV protects against ischemic brain injury in a murine model of transient focal ischemia. *Neuroscience Letters*.

[13] Cheng C.-Y., Yao C.-H., Liu B.-S., Liu C.-J., Chen G.-W., Chen Y.-S. (2006). The role of astragaloside in regeneration of the peripheral nerve system. *Journal of Biomedical Materials Research Part A*.

[14] Zhong J., Cao H., Chen Z., Zhou F., Tan X. (2013). Wnt signaling pathways participate in Astragalus injection-induced differentiation of bone marrow mesenchymal stem cells. *Neuroscience Letters*.

[15] Miltiadous P., Kouroupi G., Stamatakis A., Koutsoudaki P. N., Matsas R., Stylianopoulou F. (2013). Subventricular zone-derived neural stem cell grafts protect against hippocampal degeneration and restore cognitive function in the mouse following intrahippocampal kainic acid administration. *Stem Cells Translational Medicine*.

[16] Iqbal K., Kazim S. F., Bolognin S., Blanchard J. (2014). Shifting balance from neurodegeneration to regeneration of the brain: a novel therapeutic approach to Alzheimer’s disease and related neurodegenerative conditions. *Neural Regeneration Research*.

[17] Park D., Lee H. J., Joo S. S. (2012). Human neural stem cells over-expressing choline acetyltransferase restore cognition in rat model of cognitive dysfunction. *Experimental Neurology*.

[18] Yu D., Silva G. A. (2008). Stem cell sources and therapeutic approaches for central nervous system and neural retinal disorders. *Neurosurgical Focus*.

[19] Hitoshi S., Alexson T., Tropepe V. (2002). Notch pathway molecules are essential for the maintenance, but not the generation, of mammalian neural stem cells. *Genes and Development*.

[20] Magnusson J. P., Göritz C., Tatarishvili J. (2014). A latent neurogenic program in astrocytes regulated by Notch signaling in the mouse. *Science*.

[21] Tanveer R., Gowran A., Noonan J., Keating S. E., Bowie A. G., Campbell V. A. (2012). The endocannabinoid, anandamide, augments notch-1 signaling in cultured cortical neurons exposed to amyloid-*β* and in the cortex of aged rats. *The Journal of Biological Chemistry*.

[22] van Tetering G., Vooijs M. (2011). Proteolytic cleavage of Notch: ‘HIT and RUN’. *Current Molecular Medicine*.

[23] Hai-yan H., Guo-qin J., Xue-li Z. (2014). Effects of Astragaloside Ⅳ on the proliferation and differentiation in neural stem cells of A*β*
_1–40_ injured embryo mouse. *China Journal of Traditional Chinese Medicine and Pharmacy*.

[24] Chen N., Zhang Q., Du Y., Chen G.-G., Zhu L.-L. (2006). Pharmacokinetics and tissue distribution of astragaloside IV in rats. *Chinese Journal of Bioprocess Engineering*.

[25] Reynolds B. A., Weiss S. (1992). Generation of neurons and astrocytes from isolated cells of the adult mammalian central nervous system. *Science*.

